# Photosensitizer Adhered to Cell Culture Microplates Induces Phototoxicity in Carcinoma Cells

**DOI:** 10.1155/2013/549498

**Published:** 2012-12-23

**Authors:** Verena Ziegler, Tobias Kiesslich, Barbara Krammer, Kristjan Plaetzer

**Affiliations:** ^1^Laboratory of Photodynamic Inactivation of Microorganisms, Department of Materials Science and Physics, University of Salzburg, Hellbrunnerstraße 34, 5020 Salzburg, Austria; ^2^Department of Molecular Biology, University of Salzburg, Hellbrunnerstraße 34, 5020 Salzburg, Austria; ^3^Department of Internal Medicine I, Paracelsus Medical University and Salzburger Landeskliniken (SALK), Muellner Hauptstrasse 48, 5020 Salzburg, Austria; ^4^Institute of Pathology, Paracelsus Medical University and Salzburger Landeskliniken (SALK), Muellner Hauptstrasse 48, 5020 Salzburg, Austria

## Abstract

In vitro experiments in plastic receptacles are the basis of characterization of new photosensitizers (PSs) for the photodynamic therapy. We recently reported that lipophilic PSs adhere to cell culture microplates in a kinetic-like manner (Engelhardt et al., 2011). In the current study, we examined the interaction and phototoxic effects of the microplate-adhered PS in cancer cells. Therefore, we preloaded microplates with hypericin, Foscan, PVP-hypericin, or aluminum (III) phthalocyanine tetrasulfonate chloride (AlPCS_4_) for 24 hours and measured the PS distribution after addition of A431 human carcinoma cells: following another 24 hours up to 68% of hypericin were detected in the cell fraction. The hydrophilic PVP-hypericin and AlPCS_4_ also diffused into the cells, but the quantities of PS adherence were considerably lower. Microplate-adhered Foscan appeared not to be redistributed. In contrast to the hydrophilic PSs, the cellular phototoxicity of microplate-adhered lipophilic PS was high, independent of whether the PS (i) was pre-loaded onto microplates or (ii) added simultaneously with the cells or (iii) one day after cell seeding. Based on these results, we suggest testing lipophilic PS dyes for their adherence to microplates. Furthermore, the ability of plastic materials to (reversibly) store PSs might represent a new approach for the PS delivery or the development of antimicrobial coatings.

## 1. Introduction

In the past decades several photodynamic approaches based on a photosensitizing agent and its interaction with visible light have gained attention. The most prominent and successful one is photodynamic therapy (PDT), a well-established treatment for the eradication of neoplastic cells (for review see [1–3]). Up to date, approvals for the application of several photosensitizers against malignant and nonmalignant indications exist in Europe, the US, Canada, and Japan [4]. Also, photodynamic inactivation of microorganisms (PDI) has become very important due to the fact that even antibiotic-resistant bacteria are susceptible to this treatment [5, 6] and that natural substances may be employed as photoantibiotics [7]. Both PDT and PDI are based on the (semi-)selective uptake of a photosensitizing agent, the photosensitizer (PS), into the target tissue or cells and subsequent illumination with visible light. This results in the generation of reactive oxygen species (ROS)—most importantly singlet oxygen—thereby eradicating target cells without harming healthy tissue [4].

Cell-based in vitro experiments are a key tool not only in PDT/PDI research but generally for the development and improvement of anticancer or antimicrobial drugs. Such cell culture studies offer several advantages such as cost-efficiency, high throughput (compared to animal testing), no ethical objections, and an extraordinary flexibility. However, the limitations of cell culture experiments include most importantly the limited transferability of in vitro testing to the in vivo situation. Also PDT/PDI research frequently struggles with this constriction, in particular regarding the dosimetry of PSs and incubation parameters. 

As PDT and PDI are a multiparametrical procedures—especially with newly synthesized or discovered PSs—in vitro testing in the standard (96-well) microplate format (MP) is routinely performed to screen for typical parameters such as cellular uptake kinetics, duration, and intensity of illumination, PS concentration, and incubation times. Plastic microplates are widely used in these initial experiments due to the convenient handling and the possibility of multiparametric analyses. Nevertheless, the plastic material, usually polystyrenes or polypropylenes, may interact with the molecules of interest and influence biological processes and results. 

In a previous study we demonstrated the adherence of PSs to the surface of plastic microplates and systematically analyzed the resulting background fluorescence and its influence on measurement results regarding cellular PS uptake data. We identified differences between PSs with different physiochemical properties, but also between microplates of different manufacturers and illustrated the need of controls to correct for fluorescence caused by adhering PS [8]. 

In this study we focus on the cytotoxic effects of plate-adhered PS in photodynamic experiments. Four well-established PSs with different physicochemical properties such as lipophilicity were used to investigate the uptake of plate-adhered PS into cells and its phototoxic effect upon illumination. In continuance to our previous report [8] A431 human epidermoid carcinoma cells serve as a model cell line, due to their frequent use and wide distribution, including PDT-related research and their advanced characterization (e.g., see [9–11]). PSs were selected in order to represent a wide spectrum of available PSs and based on our findings by Engelhardt et al. [8]. Foscan is a clinically approved and widespread bacteriochlorin with a strong lipophilic character; hypericin represents a group of naturally occurring PSs, offering a high fluorescence quantum yield and reduced photobleaching [12]. Including polyvinylpyrrolidone- (PVP-) hypericin allows for the direct comparison between the hydrophobic PS hypericin and a water-soluble formulation of this compound. The phthalocyanine AlPCS_4_ (aluminum (III) phthalocyanine tetrasulfonate chloride) was tested to represent a group of strongly hydrophilic PSs. As a result of the high adherence of lipophilic PSs to Costar MPs [8] we used these plates for all the experiments. Furthermore, we compared the resulting phototoxicity to standard PDT protocols and examined the influence of plate-adhered PS on cells without illumination (dark toxicity). Besides demonstrating the significant photodynamic effect of plate-adhered PS, this study might open the door towards a new method of in vitro photosensitizer drug delivery in PDT, employing the property of certain PSs to adhere to nonpolar surfaces and therefore enabling localized long-term incubation provided by adhered PS.

## 2. Materials and Methods

### 2.1. Microplates

To analyze the distribution and uptake of the adherent PS into cells, 96-well clear flat bottom polystyrene plates obtained from Costar (catalog number 3598, Corning B.V. Life Sciences, Amsterdam, The Netherlands) were used. Phototoxicity of PS that adheres to microplates was investigated using 96-well plates with black walls and clear bottom (Costar, catalog number 3603).

### 2.2. Photosensitizers

Foscan (meso-tetrahydroxyphenyl chlorine, mTHPC, temoporfin) was obtained from Biolitec AG (Jena, Germany). The stock solution of Foscan was used as provided by the manufacturer (i.e., dissolved in ethanol-propylene at a stock concentration of 5.87 mM). Hypericin was provided by Planta Natural Products (Vienna, Austria) and dissolved in dimethyl sulfoxide (DMSO) at 1.98 mM for the stock solution. Hypericin bound to polyvinylpyrrolidone (PVP-hypericin) was synthesized as described by Kubin et al. [13] to establish water solubility. Stock solutions of PVP-hypericin were prepared in DPBS (200 *μ*M; Dulbecco's phosphate-buffered saline). AlPCS_4_ was obtained from Porphyrin Products (Logan, USA). For stock solutions, AlPCS_4_ was prepared in DPBS (1.0 mM).

All PS working solutions were prepared in cell culture medium without serum and all working steps that involved PS were performed under subdued light conditions.  Table 1 summarizes the PS concentrations and the detailed parameters for fluorescence measurement of each PS.

### 2.3. Cell Culture

A431 human epidermoid carcinoma cells (ATCC-Nr. CRL-1555) were cultured in Dulbecco's modified Eagle's medium (DMEM) supplemented with 10 mM HEPES, 5% (v/v) fetal bovine serum (FBS), 2 mM L-glutamine, 1 mM Na-pyruvate, 100 U mL^−1^ penicillin, and 0.1 mg mL^−1^ streptomycin. All media and supplements were obtained from PAA (Pasching, Austria). Cells were grown at 37°C and 5% CO_2_ in a humidified atmosphere; all experiments were done using cells of passage number 5 to 23.

### 2.4. PS Redistribution of Microplate-Adhered PS after Addition of Cells

**Figure 1:**
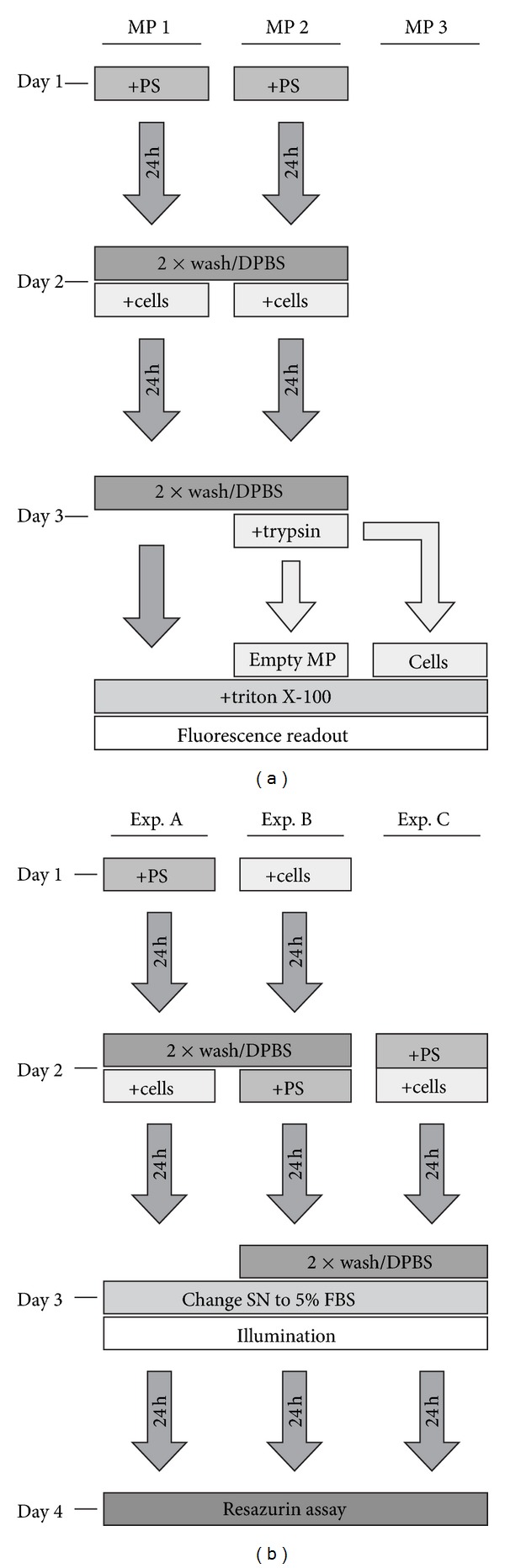
Experimental schemes. (a) The distribution of PS from microplates that were preincubated with PSs and then incubated with A431 cells was analyzed in three microplates (MP 1, MP 2, and MP 3). (b) Different incubation protocols were applied prior to photodynamic treatment to assess the phototoxicity of microplate-adhered PS. Abbreviations: DPBS: Dulbecco's phosphate-buffered saline; FBS: fetal bovine serum; MP: microplate; PS: photosensitizer; SN: supernatant.

To analyze the amount of PS that is taken up by cells after the microplate was loaded with PS, we measured PS fluorescence of cells separately from the microplate after incubation with cells (experimental scheme shown in Figure 1(a)). Therefore, the dye was diluted in DMEM without serum to the working concentrations, 100 *μ*L of medium with PS was put into a microplate and incubated for 24 h at 37°C. The microplate was then washed twice with DPBS and 0, 1·10^4^, 2·10^4^ or 4·10^4^ cells in medium without serum were added to each well. After incubation for 24 h, the medium was removed and cells were trypsinized from the microplate and transferred into a new microplate. To both microplates, the original one (“MP2”) and the one the cells were transferred into (“MP3”), 100 *μ*L 1% Triton-X 100 in DPBS was added for cell lysis 10 min prior to fluorescence intensity measurement. Additionally, in a control plate, cells were directly lyzed in the incubation plate without trypsinization (“MP1”). Fluorescence intensity was thereafter read using an Infinite M200 Pro microplate reader (Tecan, Groedig, Austria).

### 2.5. Phototoxicity of PS Adhered to Microplates

To assess the phototoxic effect of PS that is attached to microplates, we performed phototoxicity experiments with three different PS incubation protocols referred to as “Exp. A, B, and C” as illustrated in Figure 1(b).

**Exp. A**

Microplates were incubated with PS in 100 *μ*L of medium without serum for 24 h at 37°C. Afterwards, plates were washed twice with 100 *μ*L DPBS and 1·10^4^ cells were seeded into each well in medium without FBS. After another 24 h incubation, the medium was replaced by culture medium with 5% serum and cells were illuminated, according to the illumination parameters given in Table 2.

**Exp. B**

Ten-thousand cells were seeded into a microplate in medium without FBS. After 24 h incubation, the medium was changed to medium with PS and plates were again incubated for 24 h. Thereafter, cells were washed twice and culture medium with 5% serum was added before illumination.

**Exp. C**

Ten-thousand cells were seeded into microplates in medium without serum but with PS at the appropriate working concentration. Plates were incubated for 24 h and washed twice with 100 *μ*L DPBS and medium with 5% FBS was put into each well prior to illumination.

The cells were illuminated from below the cell culture receptacles using diode arrays described in Pieslinger et al. [14], with wavelengths appropriate for the according PS. After illumination, all microplates were processed identically: 24 h after illumination cellular survival was assessed by means of reduction of blue resazurin (Sigma Aldrich, Vienna, Austria), to resorufin, yielding pink fluorescence. This reaction is most probably catalyzed by diaphorases in living cells [15]. For this purpose, 20 *μ*L of resazurin (2.5 mM in DPBS) were added to each well and incubated for 2 h. The resulting fluorescence signal was measured at *λ*_ex_ = 535 nm/*λ*_em_ = 588 nm using the Infinite M200Pro microplate reader.

Based on these results, the LD50 values were calculated from the curves by interpolation. The LD50 values represent the light dose which induces 50% lethality of cells.

### 2.6. Toxicity without Illumination

To investigate the dark toxic effects of the dye, that is, without illumination, towards cells treated with a standard PDT protocol and cells seeded into preincubated plates, the viability of cells without illumination was analyzed (according to incubation protocols A and B, described above). The effects of PS incubation where then assessed by comparing cell viability to that of untreated controls.

### 2.7. Data Processing and Statistics

Mean values from microplate replicates were corrected for blank values (wells treated as described but without cells and PS). All data represent mean values of at least three independent experiments ± SEM.

## 3. Results

### 3.1. PS Redistribution of Microplate-Adhered PS after Addition of Cells

In order to determine a redistribution of PS adhered to empty microplates into eukaryotic cells after their addition, we performed PS uptake experiments to analyze the distribution of PS in cells and plates. 

For means of comparison of the basal adherence of different PSs to MPs, we calculated the ratio of the signal of wells without cells (and preloaded with PS) to blanks (no cells, no PS; see Table 3). As the blanks are supposed to exhibit a stable base fluorescence signal, this ratio is intended to show the differences in signal intensity for all PSs independent from diverging measurement parameters for the different PSs. This ratio shows that, for 1.0 *μ*M hypericin, the total fluorescence is 5.30-fold higher in controls w/o cells compared to blanks; for 5.0 *μ*M hypericin, the signal intensity ratio almost doubled to a factor of 9.26. Furthermore, for all PSs analyzed a 5-fold increase in PS concentration does not result in an equally increased fluorescence level in the controls, but only in an elevation in between a factor of 1.43 and 1.86 compared to the lower PS concentration.

**Figure 2:**
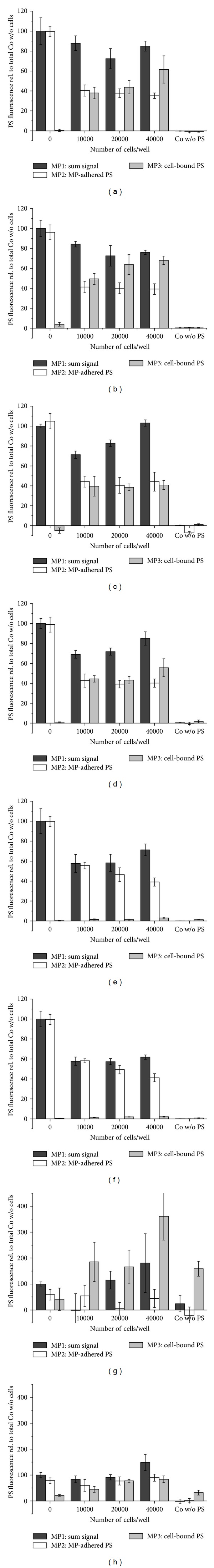
Distribution of microplate-adhered PS after addition of cells. The distribution of PS from microplates was determined by plates that were preincubated with 1.0 *μ*M hypericin (a) or 5.0 *μ*M hypericin (b); 1.0 *μ*M PVP-hypericin (c) or 5.0 *μ*M PVP-hypericin (d); 0.4 *μ*M Foscan (e) or 2.0 *μ*M Foscan (f) or 10 *μ*M AlPCS_4_ (g) and 50 *μ*M AlPCS_4_ (h). The amount of PS was assessed directly in the incubation plate (MP1) or cells were transferred from the incubation microplate to a new microplate to analyze the PS remaining adhered to the plate (MP2) or having been bound to cells (MP3). Parameters for fluorescence intensity measurements are listed in Table 2. Results are displayed relative to controls preincubated with PS but without cells. Abbreviations: MP: microplate; PS: photosensitizer.

Under cell-free conditions and for all PSs and concentrations employed, no significant percentage of PS was detached from the plastic material, as no fluorescence was detected in the according control wells of the new MP (“MP3,” Figure 2). After incubating the MPs for 24 h with 1 *μ*M hypericin (Figure 2(a)) or 5 *μ*M hypericin (Figure 2(b)), the highest concentration of the lipophilic hypericin was found in samples where no cells have been added. If cells were added after 24 h PS incubation, for both hypericin concentrations, the total amount of PS decreased (“MP1”). Compared to wells without cells, constantly around 40% of hypericin remained adhered to the MP after removal of the cells by trypsinization (“MP2”). When analyzing these separately measured cells (“MP3”), we found that 37%–67% of hypericin has been taken up by cells; the total amount of cell-bound hypericin increases with increasing cell numbers per well.

For MPs that were preincubated with the water-soluble PVP-hypericin (Figures 2(c) and 2(d)) in samples with 10000 or 20000 cells up to 30% of the PS was lost if cells were lysed directly in the plate (“MP1”). Again about 40% of the remaining PS adheres to the MP (“MP2”), whereas, for the lower concentration of 1.0 *μ*M, a slightly lower rate was internalized into the cells and detected in the separate MP (“MP3”). For 5.0 *μ*M PVP-hypericin, even a higher amount of up to 55.6% of the PS was found to be moved to the new microplate with the cells. Taking into account the control/blank ratio (Table 3), we can also see that, compared to pure hypericin, in total by far less PVP-hypericin adheres to the MP after 24 h incubation. The fluorescence in the control is only 1.94-fold higher than the blank for 1 *μ*M PVP-hypericin and 2.77-fold higher for 5 *μ*M, respectively. 

After incubation of microplates with 0.4 *μ*M (Figure 2(e)) or 2.0 *μ*M (Figure 2(f)) of the lipophilic PS Foscan, 29%–61% of the PS are lost by addition of cells (“MP1”); the remaining PS is almost completely adhered to the MP (“MP2”). Only a small amount of PS, not exceeding 3% of fluorescence measured in PS-control wells, is found to be transferred to the new plate together with the cells (“MP3”). These values are stable and seem independent from the cell number and Foscan concentration, although we observe a slight trend of decreasing rates of plate-adhered PS with increasing cell numbers per well. In total, the amount of plate-adhered PS is extremely high for this PS, as the fluorescence in the controls w/o cells is 618.62-times higher than in the blanks after 24 h incubation with 0.4 *μ*M and elevated even by a factor of 1148.54 after incubating with 2.0 *μ*M Foscan. 

For hydrophilic AlPCS_4_ these ratios are different; the controls incubated with AlPCS_4_ exhibit only 1.10-fold the fluorescence of the blanks for 10 *μ*M and 1.79-fold for 50 *μ*M, respectively. This indicates that only very small amounts of AlPCS_4_ adhere to the MPs. For the concentration of 10 *μ*M (Figure 2(g)) it is difficult to identify clear trends due to the high experimental error caused by the low signal intensity. However, 10 *μ*M AlPCS_4_ is the only setup showing a higher fluorescence in wells containing cells than wells without cells. If cells were lysed directly in the wells preincubated with AlPCS_4_, the fluorescence of samples with 20000 or 40000 cells (“MP1”) is 115% and 180%, respectively, of controls without cells; and the effect is even more pronounced if cells were trypsinized and analyzed separately from the original MP, showing 165% to 361% fluorescence, compared to the control. For 50 *μ*M AlPCS_4_ (Figure 2(h)), once more only little amounts of PS adhere to the MP after 24 h incubation (“MP1”); therefore also the quantity of PS that is available for cells is limited. Only in this experimental setup the fluorescence of AlPCS_4_ is higher in samples with cells directly lysed in the plate than in samples without cells. In case cells are removed from the MP loaded with PS, approximately a half of the sensitizer is relocated to the second plate; the same amount is left in the original MP.

### 3.2. Phototoxicity of PS Adhered to Microplates

The effect of plate-adhered PSs on cell viability after photodynamic treatment was investigated and compared to the established in vitro PDT protocol and to a protocol with the PS being added at the same time as cells were seeded into the MPs (see Figure 1(b) for an overview). 

**Figure 3:**
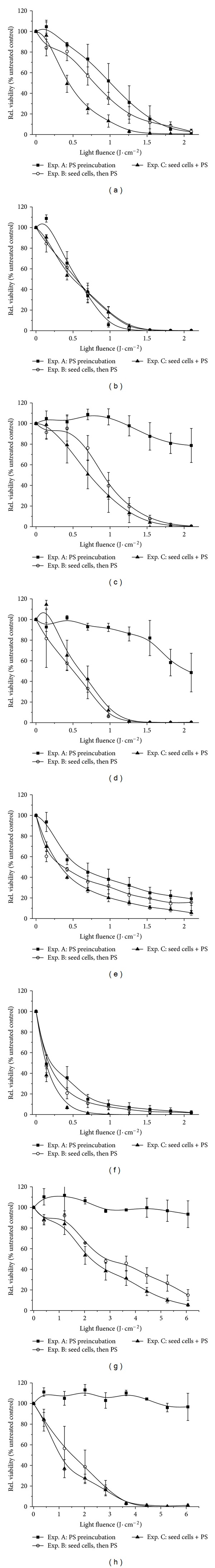
Phototoxicity of PS adhered to microplates. To determine the phototoxic effect of PS adhered to microplates, three different incubation protocols were applied; PDT protocol A with cells being seeded after plates were preincubated with PSs; the standard PDT protocol B with cells being seeded prior to PS incubation, and protocol C with cells and PS being added to the plate concurrently. As photosensitizers 1.0 *μ*M hypericin (a) or 5.0 *μ*M hypericin (b); 1.0 *μ*M PVP-hypericin (c) or 5.0 *μ*M PVP-hypericin (d); 0.4 *μ*M Foscan (e) or 2.0 *μ*M Foscan (f) or 10 *μ*M AlPCS_4_ (g) and 50 *μ*M AlPCS_4_ (h) were employed. Cell viability was determined using the resazurin assay 24 h after illumination; results are shown relative to untreated controls. Abbreviations: Exp.: experiment; PS: photosensitizer.

For 1 *μ*M hypericin (Figure 3(a)), a phototoxic effect can be observed for all three protocols as, after illumination with a light fluence of 2.1 J·cm^−2^, less than 3% of cells survived the treatment. When hypericin was added at the time of cell seeding and was incubated together with the cells, the clearest decline in viability is found with less than 5% surviving cells after illumination with 1.3 J·cm^−2^ and a 50% survival rate (LD50, Table 4) at 0.4 J·cm^−2^. The phototoxic effect is less pronounced after standard PDT (“Exp. B”) but still results in complete eradication of the cell population. An almost linear but smaller decrease of viable cells is found if only plate-adhered hypericin was available, with an LD50 of 1.0 J·cm^−2^; although even with this protocol, cells completely deceased after the highest light dose was applied. After the hypericin concentration was increased to 5 *μ*M (Figure 3(b)), the differences between the three tested PDT protocols became almost imperceptible. The LD50 values for all three protocols lie between 0.5 and 0.6 J·cm^−2^ with less than 5% survival at 1.3 J·cm^−2.^

The water-soluble formulation of hypericin, PVP-hypericin, shows a completely different behavior in terms of phototoxicity of MP-adhered PS (Figure 3(c)). Both protocols with 1 *μ*M PVP-hypericin being directly available for cells due to its addition to the incubation medium are comparable with a distinct phototoxic effect yielding LD50s of 0.9 J·cm^−2^ (“Exp. B”) and 0.7 J·cm^−2^ (“Exp. C”), and without viable cells after illumination with >1.5 J·cm^−2^. In contrast, if PVP-hypericin was preincubated with the plate (“Exp. A”), only a small reduction in cell viability can be found, with 21% toxicity after maximum illumination. With a higher PVP-hypericin concentration of 5 *μ*M (Figure 3(d)), the phototoxic effect is intensified for cells directly incubated with the PS, with a fast decrease of viability for illumination up to 1.3 J·cm^−2^ and no viable cells at higher fluences. For indirect PVP-hypericin administration via plate-adhered PS, the viability was similarly reduced to a minimum of 49% after illumination with 2.1 J·cm^−2^.

If Foscan was used as PS, the differences in response to photodynamic treatment between the three applied protocols were least distinct. With the lower concentration of 0.4 *μ*M (Figure 3(e)), the strongest phototoxic effect was achieved after simultaneously seeding and incubating the cells with Foscan. Here, we observed a continuous decline of cellular viability down to 5.6% after maximum illumination. With the standard PDT protocol, the curve progression is comparable but with a less intense effect and a final viability of 15.7%. Illumination of cells in Foscan-preincubated plates shows similarly a strong phototoxic effect, resulting in 19.6% survival after the highest light dose was applied and, compared to the other tested protocols, the highest LD50 of 0.6 J·cm^−2^. The three curves are assimilated when the Foscan concentration was increased to 2 *μ*M (Figure 3(f)). Here we found a nearly exponential decrease in cell viability for all protocols with LD50 values between 0.1 and 0.2 J·cm^−2^ and less than 5% surviving cells after illuminating with 1.6 J·cm^−2^. 

For 10 *μ*M AlPCS_4_ (Figure 3(g)), both protocols with directly available PS (B and C) show a comparable, nearly linear decrease of cell viability (although compared to all other PSs employed, the light dose has been increased up to a maximum of 6.1 J·cm^−2^). For the standard PDT-protocol, the LD50 was 2.9 J·cm^−2^ with a minimum of 15.0% remaining viable cells and an LD50 of 2.3 J·cm^−2^ and 5.4% survivors for protocol C, respectively. In contrast, if the cells were seeded into plates that were preincubated with AlPCS_4_, there was no phototoxic effect observable. After increasing the AlPCS_4_ concentration to 50 *μ*M the cytotoxic effect on cells treated with protocols B and C, that is, with direct access to PS, increased. Cell survival decreases linear, but, now with a more pronounced decline, LD50 values of 1.5 J·cm^−2^ (B) and 1.1 J·cm^−2^ (C) and almost no viability after illumination with >3.6 J·cm^−2^. Similar to the lower concentration of AlPCS_4_, for cells in preincubated plates there was no significant effect detectable. Even after maximum illumination still 96.7% of cells were intact.

### 3.3. Cytotoxicity of PS without Illumination (Dark Toxicity)

To assess the influence of the PS on viability of cells without illumination and possible differences between a standard PDT protocol and the use of PS-coated microplates, we analyzed the dark cytotoxicity. 

**Figure 4:**
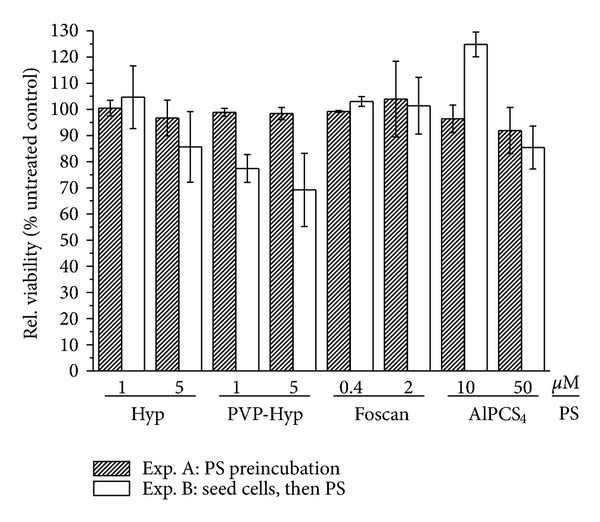
Cytotoxicity of PS without illumination. The cytotoxic effect of the four tested PSs in the dark was determined for cells seeded into microplates that were preincubated with PS and for cells that were seeded into microplates before PS was added (standard protocol). Twenty-four hours thereafter, cellular survival was assessed by means of resazurin assay. Results are expressed as ratio relative to untreated control. Abbreviations: exp, experiment; PS, photosensitizer; (PVP-)Hyp, (polyvinylpyrrolidone-)Hypericin.

For all sensitizers and concentrations tested, no dark toxic effect is notable if the cells were added to plates preincubated with the PS (Figure 4). The viability of the cells is in a range between 92% and 104% for all PSs. In the standard PDT protocol where preseeded cells were incubated with the PS afterwards, the higher hypericin concentration of 5 *μ*M caused a small but negligible increase of dark toxicity, whereas for the lower concentration cells further seem unaffected by the PS. For PVP-hypericin a decline in viability is already visible for the low concentration of 1 *μ*M and even more pronounced for 5 *μ*M (77% and 69% viability, respectively) if incubated with the standard protocol.

For Foscan, the differences in the dark toxicity between the two protocols are marginal; similarly, cells directly incubated with the sensitizer do not show decreased viability. After direct incubation of the cells with 10 *μ*M AlPCS_4_ there is a surprising increase in viability of about 25% compared to the control. In contrast, the high AlPCS_4_ concentration leads to a small but insignificant decline in survival.

## 4. Discussion

The development of a new PS from its synthesis, physicochemical, and in vitro characterization to clinical use is a multistep process. Especially the initial screening for suitable substances is based on experiments performed with cell cultures. In vitro experiments are commonly used to characterize a PS in terms of phototoxicity, effects of the PS in the dark, the uptake behavior into cells, and also the mechanisms of cell death or pathways and factors leading to successful destruction of the target cells. Basic research is reliant to this cheap and versatile tool. Within the last decades the application of single-use plastic materials has been established, allowing for high throughput analysis of key features of a PS, combined with convenient and safe handling. Experiments performed in microplates offer several convincing advantages such as the possibility of multiparametric experiments under reproducible conditions, saving time, material, and resources and therefore the improvement of the workflow. 

In a recent study we have shown several challenges concerning the use of microplates in PDT experiments. In the absence of serum lipophilic PSs adhere to standard cell culture microplates obtained from various manufacturers in a kinetic-like, time-dependent manner. This may lead to misinterpretation of MP-based experiments on cellular PS uptake if proper controls are not included [8]. 

In the current study we addressed the question whether plate-adhered PS is transferred into cells in plates that were preloaded with PS. For the lipophilic substances hypericin and Foscan we confirm our pervious observation that high amounts of the PS adhere to microplates in the absence of serum. Only very small quantities of the hydrophilic PVP-hypericin and AlPCS_4_ stick to the surfaces of MPs. In all cases we observed that addition of cells decreased the overall content of PS; to a certain extent we suppose that this is caused by loss of cells, and therewith also PS, due to washing or other mechanisms of fluorescence loss, for example, due to PS aggregation.

When we addressed the redistribution of MP-adhered PS after addition of cells, we found that, interestingly, both lipophilic photosensitizers, hypericin and Foscan behave very differently. After addition of cells to MPs preloaded with hypericin, a significant amount of the PS is redistributed and bound to the eukaryotic cells, as shown by separate fluorescence measurements of detached cells: 37%–68% of the MP-adhered hypericin relocalizes into A431 cells after addition. PVP-hypericin shows very similar properties; also, the fluorescing compound has the ability to partially detach and is incorporated into cells. However, due to a much lower adherence of the water-soluble PVP-hypericin when compared to hypericin (we observed a three-times less adherence of PVP-hypericin in average), the absolute amount of PVP-hypericin re-localizing to cells is also lower. The comparable behavior may be explained by the chemical properties of PVP-hypericin: in this formulation hypericin is not covalently bound to polyvinylpyrrolidone, but forms complexes consisting of two PVP- and one hypericin molecules [13]. As a result, a small amount of hypericin molecules might be able to adhere to the surface and then redistribute similar to pure hypericin. We hypothesize that for both hypericin-based PSs the process of redistribution is driven by the lipophilicity of the photoactive compound. Hypericin favors incorporation in the cells' lipophilic structures, such as the plasma membrane and/or membranes of the endoplasmic reticulum [16, 17]. Even though the experimental schedule of adding the PS before cells might sound hypothetical, we suggest that preloaded plastic surfaces might represent a reversible depot of hypericin. Although speculative regarding the feasibility and efficiency, plastic material—for example, particles or needles—loaded with the PS directly inserted into tumors might represent a new approach of delivering this photosensitizing substance to the target tissue without the drawback of systemic photosensitization. 

Among all PSs in this study, Foscan shows the strongest adherence to cell culture MPs. Only very small fractions of this PS diffuse into cells upon addition to preloaded MPs. However, the overall amount of MP-adhered Foscan is much higher than all other PSs tested in this study (see Table 3). Combined with the excellent phototoxicity of this substance, the relocalized PS might be sufficient to induce cell killing upon illumination. The amounts of AlPCS_4_ are too small to allow for quantitative analysis of PS redistribution. The fluorescence values are within the experimental error. 

The question whether or not the PS adhered to MPs is phototoxic to cells upon illumination is of prime importance for the comparison of cell culture-based experiments performed in plastic compartments to the in vivo situation. Our results clearly demonstrate that both lipophilic PSs are considerably phototoxic if adhered to the surfaces of microplates. At the lower concentrations employed in this study (1 *μ*M hypericin, 0.4 *μ*M Foscan) the cytotoxicity of the PSs preloaded to microplates is only slightly lower when compared to either adding the respective photoactive substance to the cells or seeding out cells in medium with PS. Surprisingly, at 5-fold higher concentrations (5 *μ*M hypericin, 2 *μ*M Foscan) the differences among all three experimental procedures are minimized and preloading MPs with either of both substances is comparably phototoxic as the standard PDT procedure. We cannot distinguish whether the PS re-localizing/binding to target cells (see Figure 1) or the photo-activated dye adhered to the MP (or a combination of both) causes the lethal overproduction of reactive oxygen species. However, loading plastic surfaces with PSs such as Foscan could further be contemplated in order to accomplish disinfection of these surfaces from multiresistant bacteria, although the efficacy of this application on prokaryotic cells has not been verified yet. 

Preincubation of MPs with either PVP-hypericin or AlPCS_4_, followed by the addition of A431 cells does not induce a significant phototoxic effect to this model system. Cell death can only be observed in case the PSs are either added after or in combination with seeding of cells. Five *μ*M PVP-hypericin and relatively high light fluences (>2 J·cm^−2^) cause 50% of cells to lose their metabolic activity, which is not the case for 50 *μ*M AlPCS_4_. Both substances differ in their hydrophilic character: AlPCS_4_ is hydrophilic *per se*; PVP-hypericin is made water soluble by the addition of polyvinylpyrrolidone to hypericin [13], resulting in a different behavior at the higher concentrations employed.

Concluding, lipophilic photosensitizers, such as hypericin and Foscan, adhere to the surface of microplates and have the potential to at least partially relocalize into cells. Upon illumination, these dyes induce a phototoxic effect comparable to the standard in vitro PDT procedure. For the hydrophilic molecules PVP-hypericin and AlPCS_4_, the small amounts of plate-adhered PS are not sufficient to induce cytotoxicity in added cells when photo-activated. We suggest that lipophilic substances should be tested for their adherence to cell culture microplates in order to rule out artifacts and provide better comparability to the in vivo situation. Furthermore, the ability of these plastic materials to reversibly store PS molecules might turn out as worth of examining as a new approach for PS delivery to (bacterial) target cells or (eukaryotic) tissue or for development of antimicrobial coatings of plastic surfaces.

## Figures and Tables

**Table 1:** Photosensitizer concentrations, fluorescence measurement parameters, and physicochemical characteristics.

Photosensitizer	Concentrations (*μ*M)	Excitation wavelength (nm)	Emission wavelength (nm)	MW (g mol^−1^)	Lipophilicity
Hypericin	1.0, 5.0	340	604	504	+++
PVP-Hypericin	1.0, 5.0	340	604	504^a^	− −
Foscan	0.4, 2.0	426	658	680	+++
AlPCS_4_	10.0, 50.0	410	684	895	− − −

^a^Molecular weight of the photoactive compound hypericin.

**Table 2:** Illumination parameters for photodynamic treatment.

Photosensitizer	Illumination wavelength	Light intensity	Light fluence
	(nm)	(mW·cm^−2^)	(J·cm^−2^)
Hypericin	610 ± 10	2.33	0.0–2.1
PVP-Hypericin	610 ± 10	2.33	0.0–2.1
Foscan	660 ± 10	2.33	0.0–2.1
AlPCS_4_	660 ± 10	6.74	0.0–6.1

**Table 3:** Fluorescence intensity of wells preloaded with PS (without cells) relative to blank wells.

Photosensitizer		Ratio (preloaded wells)/(blank)
Hypericin	1.0 *μ*M	5.30
	5.0 *μ*M	9.26

PVP-hypericin	1.0 *μ*M	1.94
	5.0 *μ*M	2.77

Foscan	0.4 *μ*M	618.62
	2.0 *μ*M	1148.54

AlPCS_4_	10 *μ*M	1.10
	50 *μ*M	1.79

**Table 4:** Photosensitizing efficiency of tested PDT protocols expressed as LD50.

Photosensitizer		LD50 (J·cm^−2^)		
		Exp. A	Exp. B	Exp. C
Hypericin	1.0 *μ*M	1.00	0.79	0.44
	5.0 *μ*M	0.56	0.55	0.50

PVP-hypericin	1.0 *μ*M	n.a.	0.91	0.72
	5.0 *μ*M	2.06	0.51	0.60

Foscan	0.4 *μ*M	0.59	0.37	0.33
	2.0 *μ*M	0.20	0.17	0.14

AlPCS_4_	10 *μ*M	n.a.	2.87	2.25
	50 *μ*M	n.a.	1.52	1.05

n.a.: not applicable.

## Abbreviations

A 431: Human epithelial carcinoma cells
AlPCS_4_: Aluminum (III) phthalocyanine tetrasulfonate chloride
DMEM: Dulbecco's modified Eagle's medium
DMSO: Dimethyl sulfoxide
DPBS: Dulbecco's phosphate-buffered saline
FBS: Fetal bovine serum; Fos, Foscan
HEPES: 4-(2-Hydroxyethyl)-1-piperazineethanesulfonic acid
MP(s): Microplate(s)
mTHPC: Meso-tetrahydroxyphenyl chlorine
PDI: Photodynamic inactivation of microorganisms
PDT: Photodynamic therapy
PS(s): Photosensitizer(s)
PVP: Polyvinylpyrrolidone
ROS: Reactive oxygen species
SEM: Standard error of mean
w/o: Without.
